# Selenium intake, food sources, and associated factors in Brazilian Longitudinal Study of Adult Health (ELSA-Brasil): a cross-sectional study

**DOI:** 10.1590/1516-3180.2024.0104.R1.07032025

**Published:** 2025-05-30

**Authors:** Elen Cintia Vale Pedro, Jéssica Levy, Dirce Maria Lobo Marchioni, Isabela Judith Martins Bensenor

**Affiliations:** I Centro de Pesquisa Clínica e Epidemiológica, Hospital Universitário, Universidade de São Paulo (USP), São Paulo (SP), Brazil.; II Post-Doctoral Research, Núcleo de Pesquisa Pescado para Saúde, Universidade de São Paulo (USP), São Paulo (SP), Brazil; Instituto de Pesca (IP-APTA), Santos (SP), Brazil.; III Faculdade de Saúde Pública, Universidade de São Paulo (USP), São Paulo (SP), Brazil.; IV Faculdade de Medicina da USP, Universidade de São Paulo (USP), São Paulo (SP), Brazil.

**Keywords:** Nutritional sciences, Selenium, Eating, Socioeconomic factors, Lifestyle, Selenium intake, Food sources, Nutrient intake

## Abstract

**BACKGROUND:**

Selenium is essential to human health. There are few reports on the analysis of selenium intake in the Brazilian population; however, data have shown that Brazilian are in the deficient range of consumption.

**OBJECTIVES:**

This study aimed to identify the major foods that contribute to dietary selenium and determine the socioeconomic and lifestyle factors associated with selenium intake.

**DESIGN AND SETTING:**

This cross-sectional study used baseline data from the Brazilian Longitudinal Study of Adult Health (ELSA-Brasil).

**METHODS:**

Selenium consumption was evaluated using a food frequency questionnaire (FFQ) developed and validated for the ELSA-Brasil. To determine the contribution of selenium consumption, each food item was divided by the total selenium intake of the population. Associations between selenium intake (mg/day, dependent variable) and sociodemographic and lifestyle factors (predictors) were tested using multiple linear regression analyses.

**RESULTS:**

The sample comprised 14,780 participants, most of whom were adults (78.5%). Individuals with income ≥ 3 minimum wages were mostly concentrated in the 5th quintile of selenium consumption; positive and significant correlations were found between selenium intake and female sex, age ≥ 60 years, income ≥ 3 minimum wages, higher or postgraduate education, alcohol consumption, and moderate physical activity level. Nuts and fish contributed the most to dietary selenium.

**CONCLUSION:**

Nuts, meat, and fish contributed the most to the dietary intake of selenium, and selenium intake was associated with sociodemographic and lifestyle factors among the evaluated individuals.

## INTRODUCTION

Selenium (Se) is an essential mineral for human health; after iodine, it is the second most important nutrient for proper functioning of the thyroid.^1^ It acts as a cofactor of more than 25 selenoproteins and selenoenzymes, which are involved in diverse physiological processes.^2^ Unlike other minerals, selenium is incorporated into proteins by means of a co-translational mechanism as part of the amino acid selenocysteine (SeCys), the 21st proteinogenic amino acid in humans.^3^

Daily intake of selenium is necessary to maintain natural metabolism and homeostasis in the human body. The known biological functions of selenium in the human body include defense against oxidative stress, regulation of thyroid hormone, protection against oxidative damage, reducing the risk of chronic non-communicable diseases, and increasing resistance of the immune system in the form of selenoproteins.^4^

The Recommended Dietary Allowance (RDA) of selenium for both men and women is 55 μg/day. The Tolerable Upper Intake Level (UL) of selenium for adults is set at 400 μg/day to prevent selenosis caused by high intake of selenium.^5^ Selenium consumption is mostly dependent on the food content and dietary supplements.^6^ Fruits and vegetables, in general, have a low content of this micronutrient.^7,8,9^

The concentration of selenium in food depends on the content of selenium present in the soil in which the plants is grown; thus, the concentration of selenium in food varies worldwide. There are few reports on the analyses of selenium in Brazilian soils; however, data have shown that many soils are in the deficient range.^10,11,12,13^

Few food items such as Brazilian nuts and kidney beef are considered the best sources of selenium.^14^ In addition to these, selenium is present in beef, chicken, fish, dairy products, and eggs.^7,8,15,16^ In regions with soil containing adequate selenium, wheat represents a good source, and thus, consumption of breads and cereals can contribute to the nutrient intake.^6,17,18,19^

Many studies show that personal factors, such as sex, age, physical activity level, and socioeconomic factors based on income and education, may affect the food consumption of a person or population.^20,21^ People from lower socioeconomic background have a higher incidence of premature death, heart diseases, and cancer than those from socioeconomically advantaged groups.^22,23,24^ In Brazil, the consumption of milk, fish, lean meat, fruits, vegetables, and whole grains is positively related to family income,^25^ which may influence selenium intake in the Brazilian population.

## OBJECTIVE

Thus, this study aimed to identify the main foods that contribute to the dietary consumption of selenium and to determine the socioeconomic and lifestyle factors associated with selenium intake among participants of the Brazilian Longitudinal Study of Adult Health (ELSA-Brasil).

## METHODS

### Study design and population

This cross-sectional study used baseline data from the ELSA-Brasil, which is a multicenter cohort study focused on the incidence of cardiovascular diseases and diabetes in the Brazilian population.^26,27^ This cohort involved 15,105 participants of both sexes, aged 35–74 years at baseline, recruited between August 2008 and December 2010.^26,27^ Detailed methodological information and baseline data have been published elsewhere.^26,27^ We excluded participants without food consumption information (n = 24) and individuals below the 1st percentile and above the 99th percentile of the total energy intake estimates (n = 301) to exclude possibly invalid food intake data. The final sample comprised 14,780 participants. The ELSA-Brasil was approved by the Research Ethics Committee of each institution where the project was conducted.

### Food consumption assessment

To evaluate the habitual consumption of participants in the last 12 months, a food frequency questionnaire (FFQ) was developed and validated for the ELSA-Brasil.^28^ This semi quantitative FFQ designed by the ELSA-Brasil had 114 food items and was answered by interview. The FFQ used the Nutrition Data System for Research (NDSR) software (University of Minnesota, Minneapolis, 2010) for data analysis. Participants were asked at the end of the FFQ if they had changed their dietary intake in the past six months, and if the answer was “yes,” the participant would inform reason for changes in their dietary habits. Individuals who did not have any dietary data or who did not have a plausible daily caloric intake (< 500 or > 6,000 kcal) were excluded.

The proportion of selenium in diet was calculated based on the consumption of all foods containing this nutrient. Selenium intake was adjusted for the total energy intake using the residue method^29^ to reduce errors associated with food consumption measurements, and the total selenium intake was divided into quintiles (1st quintile = lowest intake; 5th quintile = highest intake). Energy-adjusted values were used for stratification of quantiles and linear regression analyses.

### Socioeconomic and lifestyle factors

At baseline, data was collected from interviews, anthropometric measurements, and clinical examinations.^26^ Socioeconomic and lifestyle variables were selected based on previous studies that addressed these determinants of food intake in the Brazilian adult population.^21,30^ Accordingly, we selected sex, age, schooling, income, self-reported skin color, smoking and alcohol habits, nutritional status, and physical activity level as variables in this study.^31^

The participants were classified according to sex as male and female and according to age as adult (34–59 years) and older (≥ 60 years). We categorized schooling as “complete elementary school,” “complete high school,” and “higher education or postgraduate.” To classify the family income *per capita,* we calculated it as equivalent to the average minimum wage between 2008 and 2010 (R$ 463.33) and then stratified into < 3 or ≥ 3 minimum wages. Self-reported skin color was categorized as “white” and “not white”.^32^

Alcohol consumption was measured in grams of ethanol per week as informed in the FFQ, and participants were classified into two categories: current and past alcohol intake. We evaluated smoking status using a semi-structured questionnaire on smoking habits at the time of interview and at a predetermined time in the past. Based on these questions, participants were categorized as “non-smokers,” “former smokers,” or “current smokers.”

To assess the nutritional status of participants, we considered the body mass index (BMI) calculated as weight (kg) divided by height squared (kg/m^2^) and classified according to the World Health Organization criteria defined as: low weight (< 18.5 kg/m^2^), normal weight (18.5–24.9 kg/m^2^), overweight (25–29.9 kg/m^2^), and obesity (≥ 30 kg/m^2^).^33^ Physical activity level was evaluated based on the International Physical Activity Questionnaire (IPAQ),^34,35^ and categorized as light, moderate, and vigorous physical activity level at leisure time.^36^ In this study, we considered physical activity level during leisure time only because it was better related to sociodemographic variables, such as schooling, income, sex, and age,^37^ used in epidemiological studies.^38^

### Statistical analysis

Energy-adjusted selenium consumption was stratified into quintiles to better represent dietary selenium ranking. Sociodemographic and lifestyle factors were described as frequencies and percentages according to the lowest (1st quintile) and highest (5th quintile) levels of selenium intake and sex of the participants. Pearson’s chi-square test was used to verify differences between groups.

To measure the proportion of food contributing to selenium intake, we used the methodology proposed by Block et al.^39^ Aiming to obtain the contribution of food in selenium consumption, each food item was divided by the total population selenium intake. Food items were listed as major contributors according to their contribution rankings.^40^

Associations between energy-adjusted selenium intake (mg/day, dependent variable) and sociodemographic and lifestyle factors (predictors) were tested by multiple linear regression analyses using the stepwise backward method.

The energy-adjusted selenium intake variable approaches normality based on the results of the Shapiro-Wilk test and visual inspection of histograms and Q-Q plots, thus meeting this assumption for multiple linear regression. The multivariable model was further adjusted for self-reported changes in dietary habits over the past six months (yes or no) and supplement use (no, regular, or not regular). Since we did not have information about the presence of selenium in supplements, we adjusted the multivariable model for the use of supplements. Since information regarding the use of supplements was self-reported, we cannot exclude the possibility of memory bias. All analyses were performed using the Stata^®^ (version 14) software, assuming a statistical significance level of 5%.

## RESULTS

Our sample consisted of 14,780 participants, mostly adults (78.5%), females (54.5%), non-smokers (57.1%), self-reported as white (52.5%), and with higher education status (53%). Regarding the nutritional status of the participants, 35.9% were classified as normal weight, 40.3% were overweight, and 22.9% were obese. The distribution of sociodemographic and lifestyle characteristics according to the selenium intake of all individuals is presented in Table 1 and those stratified by sex are presented in Table 2. Individuals with income ≥ 3 minimum wages as well as men and women with higher education were mostly concentrated in the 5th quintile of selenium consumption (Table 2), whereas those who self-reported as white, who practice light physical activity, who were non-smokers and alcohol consumption had selenium intake levels in the last quintile (Table 2).

**Table 1:** Distribution of sociodemographic and lifestyle characteristics of the study population according to the selenium intake quintiles. ELSA-Brasil, 2008–2010.

Characteristic	N	%	P
**Age, years**			< 0.001
*Adult*	11.598	78.47	
*Elderly*	3.182	21.53	
**Body mass index, kg/m** ^2^			< 0.001
*Less than 18.5*	139	0.94	
*18.5 to 24.9*	5.309	35.93	
*25 to 30*	5.946	40.25	
*More than 30*	3.386	22.88	
**Self-reported race**			< 0.001
*White*	7.665	52.46	
*Not White*	6.947	47.54	
**Education level**			< 0.001
*Complete primary education*	1.852	12.53	
*Complete high school*	5.094	34.47	
*Higher education or postgraduate*	7.834	53	
**Income**			< 0.001
*< 3SM*	7.370	49,86	
≥ *3SM*	7.410	50,14	
**Smoking**			< 0.001
*Non-smoker*	8.434	57.07	
*Former smoker*	4.429	29.97	
*Current smoker*	1.916	12.96	
**Alcohol intake**			< 0.001
*Past*	2.969	22.5	
*Current*	10.224	77.5	
**Leisure-time physical activity**			< 0.001
*Light*	11.437	77.04	
*Moderate*	2.029	13.93	
*Vigorous*	1.314	09.02	

Selenium intake quintiles range from 32.44 mcg/day (minimum) to 1099.34 mcg/day (maximum). Values are presented as absolute numbers (n) or percentages (%). Differences between groups were tested using the Pearson’s chi-square test. Statistical significance was set at P < 0.05.

**Table 2:** Sociodemographic and lifestyle data according to selenium intake stratified by sex. ELSA-Brasil, 2008–2010.

Characteristic	Selenium intake (quintiles)									
	Male					Female				
	1º Q		5º Q		P	1º Q		5º Q		P
**Selenium quintiles (mcg/day, min-max)**	35.77–105.04		223.06–1054.91			32.44–105.05		223–1099.34		
**Age (n, %)**					< 0.001					< 0.001
*Adult*	1.368	80.7	934	71.03		1.059	81.68	1.203	73.31	
*Elderly*	291	17.54	381	28.97		238	18.35	438	26.69	
**BMI (n, %)**					0.003					< 0.001
*Less than 18.5*	23	1.39	8	0.61		10	0.77	18	1.1	
*18.5 to 24.9*	566	34.14	472	35.92		467	36.3	719	48.81	
*25 to 30*	719	43.37	608	46.27		463	35.73	555	38.82	
*More than 30*	350	21.11	226	17.2		356	27.47	349	21.27	
**Self-reported race (n, %)**					< 0.001					< 0.001
*White*	693	42.33	879	68.14		497	38.53	997	61.35	
*Not White*	944	57.67	411	31.86		793	61.47	628	38.65	
**Education level (n, %)**					< 0.001					< 0.001
*Complete primary education*	430	25.92	80	06.08		219	16.89	68	4.14	
*Complete high school*	753	45.39	230	17.49		634	48.88	359	21.88	
*Higher education or postgraduate*	479	28.69	1.005	76.43		444	34.23	1.214	73.98	
**Income (n, %)**					< 0.001					< 0.001
*< 3SM*	1.160	69,92	359	27.3		901	69.47	476	29.01	
≥ *3SM*	499	30,08	956	72.7		396	30.53	1.165	70.99	
**Smoking (n, %)**					< 0.001					< 0.001
*Non-smoker*	777	46.84	702	53,38		778	59.98	983	59.9	
*Former smoker*	559	33.69	475	36.12		307	23.67	504	30.71	
*Current smoker*	323	19.47	138	10.49		212	16.35	154	9.38	
**Alcohol intake (n, %)**					< 0.001					< 0.001
*Past*	374	23.94	190	15		303	29.94	294	20.01	
*Current*	1.188	76.06	1.077	85		709	70.06	1.175	79.99	
**Leisure-time physical activity (n, %)**					< 0.001					< 0.001
*Light*	1.289	79.08	831	63.92		1.117	87.61	1.192	73.76	
*Moderate*	207	12.7	243	18.69		110	8.63	265	16.4	
*Vigorous*	134	8.22	226	17.38		48	3.76	159	9.84	

A chi-square test was conducted considering all quintiles of selenium intake; however, for better interpretation and data presentation, only the extremes (1st and 5th quintiles) are included in this table. The P values refer to the analysis considering all quintiles. Statistical significance was set at P < 0.05.

The top 10 foods contributing to selenium intake for all participants are described in Table 3. Nuts were the largest contributor (28.9%), followed by cooked fish (8.6%), boneless meat (7.7%), French bread (6.9%), white rice (5.7%), fried fish (3.9%), spaghetti (3.5%), whole-grain bread (2.5%), light bread (2.2%), and whole milk (0.2%) (Table 3).

**Table 3:** Main food sources of selenium intake. ELSA-Brasil, 2008–2010.

Rank	Food	PC	SD	95% confidence interval
1st	Nuts	28.95	0.82	27.35; 30.56
2nd	Cooked fish	8.63	0.1	8.43; 8.83
3rd	Boneless meat	7.66	0.08	7.52; 7.81
4th	French bread	6.97	0.06	6.85; 7.1
5th	White rice	5.72	0.04	5.65; 5.8
6th	Fried fish	3.91	0.06	3.79; 4.03
7th	Spaghetti	3.53	0.04	3.46; 3.6
8th	Whole-grain bread	2.54	0.04	2.50; 2.62
9th	Light bread	2.2	0.03	2.15; 2.59
10th	Whole milk	02.03	0.03	1.98; 2.08

Nuts include Brazilian nuts, peanuts, cashew nuts, walnuts, and almonds.

PC, percentage contribution; SD, standard deviation.

Regarding the contributing foods stratified by sex, there was a difference only from the sixth-ranked foods (Table 4). Except nutritional status, all sociodemographic and lifestyle variables investigated were dependent on selenium intake (Table 5). As shown in Table 4, positive and significant correlations were found between intake of selenium and the following factors: female sex, age ≥ 60 years, income ≥ 3 minimum wages, complete high school and higher or postgraduate education levels, alcohol consumption, and moderate and vigorous physical activity levels. In contrast, smoking and “not white” skin color were negatively associated with selenium intake (Table 5).

**Table 4:** Main food sources of selenium intake by stratified by sex. ELSA-Brasil 2008–2010

Rank	Food	PC	SD	95% confidence interval
**Men**				
1st	Nuts	26.88	1.43	24.07; 29.69
2nd	Cooked fish	8.5	0.15	8.21; 8.78
3rd	Boneless meat	8.12	0.12	7.89; 8.35
4th	French bread	7.74	0.09	7.55; 7.93
5th	White rice	6.79	0.06	6.67; 6.9
6th	Fried fish	4.31	0.09	4.13; 4.5
7th	Spaghetti	3.88	0.06	3.76; 4
8th	Bean	2.44	0.03	2.38; 2.49
9th	Whole milk	2.08	0.04	2; 2.16
10th	Whole-grain bread	1.97	0.05	1.86; 2.07
**Women**				
1st	Nuts	30.98	0.81	29.39; 32.57
2nd	Cooked fish	8.76	0.14	8.49; 9.02
3rd	Boneless meat	7.22	0.09	7.03; 7.4
4th	French bread	6.22	0.08	6.06; 6.38
5th	White rice	4.68	0.04	4.60; 4.75
6th	Spaghetti	3.18	0.04	3.10; 3.25
7th	Whole-grain bread	3.11	0.06	2.99; 3.22
8th	Light bread	2.79	0.04	2.71; 2.88
9th	Oat	2.12	0.04	2.04; 2.19
10th	Cooked chicken	2.05	0.04	1.98; 2.12

Nuts include Brazilian nuts, peanuts, cashew nuts, walnuts, and almonds.

PC, percentage contribution; SD, standard deviation.

**Table 5:** Multiple linear regression model between selenium intake and sociodemographic and lifestyle factors. ELSA-Brasil, 2008–2010.

Predictors	ß	95% confidence interval	P
Sex (reference: male)			
Female	6.01	2.51; 9.5	< 0.001
Age group (reference: adults)			
Older people	12.85	8.54; 17.16	< 0.001
Self-reported skin color (reference: white)			
Non-white	(-8.66)	(-12.31; -5.01)	< 0.001
Education level (reference: complete primary school)			
Complete high school	7.22	1.28; 13.16	0.017
Higher education or postgraduate	33.34	26.96; 39.71	< 0.001
Income per capita (reference: < 3 minimum salaries)			
≥ 3 minimum wages	23.95	19.75; 28.14	< 0.001
Smoking (reference: non-smoker)			
Former smoker	6.29	2.43; 10.16	0.001
Current smoker	(-7,77)	(-13.02; -2.53)	0.004
Alcohol (reference: non-consumer)			
Current consumption	4.38	0.19; 8.56	0.040
Nutritional status (reference: normal weight)			
Low weight	(-9.47)	(-27.67; 8.73)	0.308
Overweight	(-11.84)	(-30.04; 6.36)	0.202
Obesity	(-14.7)	(-33.07; 3.68)	0.117
Physical activity level (reference: light)			
Moderate	10.03	5.02; 15.04	< 0.001
Vigorous	15.52	9.57; 21.48	< 0.001

## DISCUSSION

In this study, we identified food sources that were major contributors to the total selenium intake, which included nuts, cooked fish, boneless meat, French bread, and white rice among ELSA-Brasil participants (2008–2010). The nuts consumed included Brazilian nuts (52.6%), peanuts (18.8%), cashew nuts (15%), walnuts (6.8%), and almonds (6.8%). Brazilian nuts represent the largest fraction of nut consumption, which justifies their high selenium concentration and consumption. These foods are part of regular food consumption of Brazilian families, except nuts, owing to their high cost, and meat in individuals from lower socioeconomic classes. In addition, nuts consumption is not common in most regions of Brazil being not a part of the Brazilian eating habits, representing only 0.2% of the total calories consumed per day.^41^ A cross-sectional population-based study aimed to identify foods contributing to mineral intake among residents in urban areas of São Paulo revealed that white rice, meat, and bread were the main foods contributing to selenium intake.^16^

Many people rarely eat foods that are good sources of selenium, such as Brazilian nuts, the richest dietary source of selenium.^7,8,13,15^ However, bread and cereals are commonly consumed and make a substantial contribution to selenium intake, followed by animal products. Ferreira et al.^42^ evaluated the selenium content of various foods consumed in the country from different regions and found that the staple foods in the Brazilian diet, such as white rice, beans, wheat flour, corn flour, and cassava flour, contained low levels of this mineral. Significant amount of selenium was present only in meat (both beef and fish). These findings indicate that the Brazilian population is susceptible to selenium deficiency, which is further aggravated among lower-income individuals as they have restricted access to animal foods.

Socioeconomic characteristics, such as sex, age, race/ethnicity, education level, and income, can influence selenium intake among the ELSA-Brasil participants. Dietary selenium intake is higher in older people than in adults, and there is a positive association between age and micronutrient consumption. This observation may reflect healthier diet consumed by older people to minimize age-related disorders, as described by Andrade.^43^

In our analysis, women had a high selenium intake, which might be due to better care of diet quality by women than men. A multi-national study including participants from 23 countries revealed that women focus more on healthy eating habits than do men, and that health beliefs explain a large proportion of dietary behavior.^44^ Several other studies conducted in Brazil and other countries also found a better dietary profile in women.^44,45,46,47^ In addition, women with normal weight (BMI: 18.5–24.9 kg/m^2^) had a high intake of dietary selenium, reflecting better diet profile adopted by women and their concern about diet quality. A similar study with the same sample from the ELSA-Brasil found a greater consumption of antioxidants, such as vitamins A and E and selenium, by females.^48^

Regarding ethnicity-based dietary differences, individuals who self-reported as “white” (52.5%) had a high intake of dietary selenium. According to our findings, selenium intake, based on the NHANES III 24-h dietary recall, was slightly higher among Whites than Blacks.^49^ Racial variations in selenium status has been reported in previous studies as well.^49,50,51,52^ Non-white population experiences significant disparities in selenium intake, which may be linked to both socioeconomic factors and lack of nutritional knowledge. Previous studies suggest that lower consumption of selenium-rich foods is prevalent among Blacks than among Whites.^49,51,54^ Selenium deficiency in this population is concerning as selenium plays a crucial role in antioxidant defense and cardiovascular health.^3,6^

Limited nutritional education and access to resources have contributed to these disparities. Many individuals in underserved communities lack awareness of their specific dietary needs, including the importance of micronutrients, such as selenium, in preventing chronic diseases and supporting mental health.^3^ This knowledge gap leads to poor dietary choices and exacerbates health risks.^24^

High school education level is associated with a higher consumption of nutrients such as selenium. In our study, individuals with higher education levels or postgraduates were mostly concentrated in the 5th quintile of selenium consumption for men and women. Consistent with our observations, a previous study by Cardoso et al.^53^ with the same ELSA-Brasil sample found that the consumption of a “healthy” diet characterized by fruit, vegetables, whole grains, and low sugar/low fat patterns were present in strikingly higher proportions among individuals with higher education levels.

Socioeconomic status has an important impact on the lifestyle of individuals by determining access to services, goods, and products, including food. Medina et al.^54^ verified a better profile of food consumption in high-income and educated social groups. In our study, individuals with higher incomes were mostly concentrated in the 5th quintile of selenium consumption for both men and women, and there was a positive association between high income and consumption of this micronutrient. Although the consumption of selenium was higher in women, not all individuals who self-reported as “white” with high education and higher income had adequate consumption of selenium because the choice of foods in the diet may be determined by other reasons, such as family or personal preferences, availability of foods near home or place of work, seasonal foods, or knowledge about healthy and unhealthy foods.

Individuals who were former smokers, moderate and vigorous physical activity, and alcohol consumption were positively associated with selenium intake. The dietary habits of smokers are usually characterized by a lower intake of antioxidants such as selenium and fiber.^55,56^ This suggests that these differences may contribute to the deleterious effects of tobacco smoke components on the risk of cancer and coronary heart disease.^56^

In the present study, alcohol consumption was significantly higher among men and women in the 5th quintile of selenium consumption, and there was a positive correlation between current alcohol consumption and selenium intake. Alcohol consumption is usually accompanied by the consumption of protein snacks, such as meat and nuts, which contribute to the intake of foods rich in selenium. Isobe et al.^57^ in a cohort study of 1,183 subjects observed that alcohol intake was associated with relatively selenium-rich foods such as seafood. The results from the NHANES 2003–2008 showed that men consumed more energy from meat and fish on drinking days than on non-drinking days.^58^

A physically active lifestyle can modify eating habits. It has been suggested that physical activity could be a possible gateway for healthier eating and better food choices.^59,60^ In our study, there was a positive correlation between light physical activity and selenium intake.

In this study, foods that contributed mostly to dietary selenium intake were nuts, meat, and fish among the individuals evaluated, and low intake of selenium was associated with the following conditions: male sex, low education, low income, non-white skin color, and younger age, indicating that the Brazilian population may be susceptible to selenium deficiency; public policies focused on the increased intake of foods rich in selenium must be directed specifically to groups with specific characteristics of low selenium intake.

## CONCLUSION

Nuts, meat, and fish contributed the most to selenium intake in the diet. In addition, selenium intake was associated with sociodemographic and lifestyle factors, such as sex, age group, education, income, ethnicity, smoking, and physical activity among the evaluated individuals.
